# Digital PCR for Single-Cell Analysis

**DOI:** 10.3390/bios14020064

**Published:** 2024-01-24

**Authors:** Weibo Fang, Xudong Liu, Mariam Maiga, Wenjian Cao, Ying Mu, Qiang Yan, Qiangyuan Zhu

**Affiliations:** 1 Research Center for Analytical Instrumentation, Institute of Cyber-Systems and Control, College of Control Science and Engineering, State Key Laboratory of Industrial Control Technology, Zhejiang University, Hangzhou 310027, China; 11632035@zju.edu.cn (W.F.); lxd112@zju.edu.cn (X.L.); mayoumi@zju.edu.cn (M.M.); wenjian.cao@zju.edu.cn (W.C.); muying@zju.edu.cn (Y.M.); 2 Department of Hepatobiliary and Pancreatic Surgery, Huzhou Central Hospital, Huzhou Key Laboratory of Intelligent and Digital Precision Surgery, Department of General Surgery, Affiliated Huzhou Hospital, School of Medicine, Zhejiang University, Huzhou 313000, China; 3 Huzhou Institute of Zhejiang University, Huzhou 313002, China

**Keywords:** digital PCR, microfluidic chip, single-cell analysis

## Abstract

Single-cell analysis provides an overwhelming strategy for revealing cellular heterogeneity and new perspectives for understanding the biological function and disease mechanism. Moreover, it promotes the basic and clinical research in many fields at a single-cell resolution. A digital polymerase chain reaction (dPCR) is an absolute quantitative analysis technology with high sensitivity and precision for DNA/RNA or protein. With the development of microfluidic technology, digital PCR has been used to achieve absolute quantification of single-cell gene expression and single-cell proteins. For single-cell specific-gene or -protein detection, digital PCR has shown great advantages. So, this review will introduce the significance and process of single-cell analysis, including single-cell isolation, single-cell lysis, and single-cell detection methods, mainly focusing on the microfluidic single-cell digital PCR technology and its biological application at a single-cell level. The challenges and opportunities for the development of single-cell digital PCR are also discussed.

## 1. Introduction

Cells are the basic units of life; however, the cells in the human body exhibit extensive heterogeneity, and many diseases come from single-cell mutation. A traditional analysis cannot clearly explain the behavior of individual cells. For example, in the case of cancer tumors, bulk analysis provides an overview of the average differences in gene expression, rather than the expression of genes in individual cells; this makes it difficult to find molecular differences which are only linked to specific cell types. Single-cell analysis can accurately provide information on intracellular substances and biochemical reactions within cells, reflecting specific relationships between cell functions and chemical components, as well as special roles for certain cells in living organisms [1].

The use of single-cell analysis is an interdisciplinary frontier field formed by the integration of analytical chemistry, biology, and medicine. It greatly promotes the understanding of life at the single-cell level and has vast applications in biomedical studies [2]. In the past decade, research on single-cell analysis has been highly favored by scholars; the number of papers published on PubMed on single-cell analysis in the past decade has shown a rapid growth trend yearly. Single-cell analysis enables scientists to study cells at the individual level, capturing the unique insights of each cell. It provides a more accurate and comprehensive picture of what happens to an organism at a specific point in time, which will help us better understand diseases. Single-cell analysis has involved in many fields, including basic research applications, such as cancer [3], immunology [4], neurology [5], stem cells [6], etc., and in clinical applications such as non-invasive prenatal diagnosis [7], in vitro fertilization [8], and circulating tumor cells (CTCs) [9].

The rapid development of single-cell analysis technology is driven by the rapid development of analytical tools such as cell separation, microfluidics, and single-cell sequencing. Microfluidic technology has been widely used for single-cell analyses [10]. The internal sizes of microfluidic devices are generally below 100 μm, and the reaction volume is typically at picoliter to nanoliter level, making them very suitable for single-cell analysis. With the development of the microfluidics, a digital polymerase chain reaction (dPCR) is a new option for single-cell analysis, and dPCR-based microfluidic devices have provided an efficient and accurate platform for single-cell analyses because of their accurate and automatic manipulation of the single-cell with high throughput and precision [11]. Digital PCR is a single-molecule amplification technique that is not applicable to the classical concept of concentration [12]. Single-molecule amplification is a stochastic issue. After sufficient amplification cycles and endpoint detection, the single molecule can be amplified efficiently, so the number of target molecules can be directly counted.

In this review, we first introduce the general process of single-cell analysis, including single-cell isolation and single-cell lysis. Next, we will briefly introduce digital PCR and its applicability for single-cell analysis. Finally, digital PCR for single-cell analysis will be summarized into two typical groups based on their working mechanisms, e.g., chamber-based digital PCR and droplet-based digital PCR.

## 2. Single-Cell Analysis

**Figure 1:**
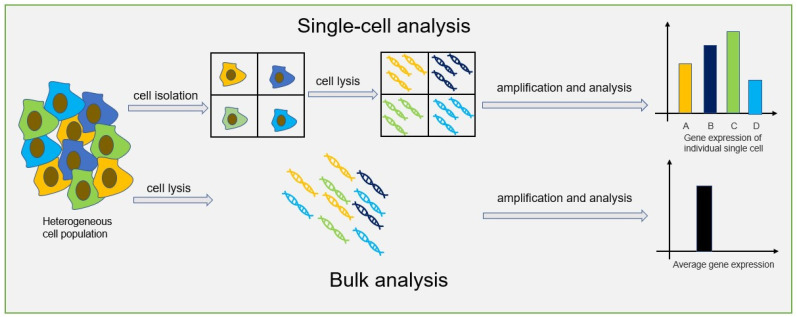
General process of single-cell analysis and bulk analysis.

It has been well-established that heterogeneity exists among cells. Traditional bulk analysis methods obtain the average value of cells, which could not clearly explain the behavior of individual cells. Compared to a bulk analysis, single-cell analysis can obtain data at single-cell level, helping researchers to better understand cellular heterogeneity, biological functions and various diseases mechanisms. The difference between single-cell analysis and bulk analysis is shown in Figure 1.

### 2.1. Single-Cell Isolation

**Figure 2:**
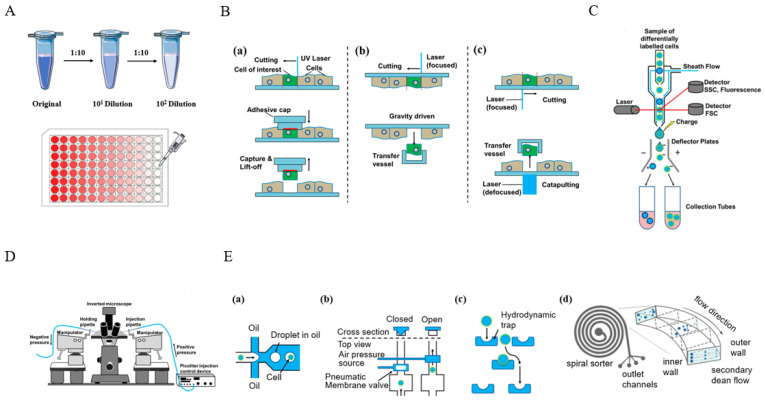
The five most-used single-cell isolation methods. (**A**) Schematic overview of limited serial dilution. (**B**) Schematic overview of laser capture microdissection (LCM) methods. (**a**) Contact-based via adhesive tapes. (**b**) Cutting with a focused laser followed by capture with a vessel. (**c**) Cutting with a focused laser followed by pressure catapulting with a defocused laser pulse. (**C**) Schematic overview of fluorescence-activated cell sorting (FACS). (**D**) Schematic overview of micromanipulator. (**E**) Schematic overview of different microfluidic methods for single-cell isolation. (**a**) Droplet-in-oil-based isolation. (**b**) Pneumatic membrane valving- based isolation. (**c**) Hydrodynamic cell traps-based isolation. (**d**) Dean flow-based isolation. Reprinted from ref. [13].

Accurate and reliable single-cell isolation from the sample population is a prerequisite for all single-cell-based methods. Consequently, researchers have developed a series of single-cell separation and isolation methods with different advantages and limitations, including limited serial dilution [13], fluorescence-activated cell sorting (FACS) [14], manual micromanipulation [15], laser capture microdissection (LCM) [16], and microfluidics methods [17], shown in Figure 2. These methods have their own characteristics, and researchers choose appropriate methods for single-cell isolation according to their needs.

Limited serial dilution [13] is a commonly used monoclonal culture method, which can also be used to obtain individual cells. The cell suspension undergoes a series of dilutions based on the distribution and concentration of cells in the cell suspension. Ultimately, only one single-cell exists in a certain volume of suspension. This method is simple in operation, low in cost, but low in efficiency because achieving a single-cell in an aliquot is statistically based on Poisson distribution.

Fluorescence-activated cell sorting (FACS) is a high-throughput cell-sorting technology based on flow cytometry. It has been used for single-cell isolation [18,19]. In FACS systems, the cell suspension which has been fluorescent labeled is pressed into the flow cell and diluted with sheath fluid to an appropriate concentration. Then, through targeted vibration driving, this stream breaks into continuous droplets, some of which carry cells. Finally, droplets containing individual cells of interest can be collected using electrically charged plates. This method has high throughput, but requires expensive equipment—flow cytometry. FACS, also, generally requires > 10,000 cells as start input, making it unsuitable for rare samples.

Laser capture microdissection (LCM) is a technology that accurately separates single cells from tissue samples [20]. After staining tissue sections, they are examined under a microscope for target area selection, and the selected area is isolated using a laser. This method does not damage the tissue structure and directly obtains target cells from frozen or paraffin-embedded tissue sections. However, this method relies on a laser capture microdissection platform, which is very complex and expensive.

Manual micromanipulation is a method of manually selecting individual cells using a microscope. Through microscopic observation, well-formed cells can be selected from the prepared cell suspension, and individual cells can be sucked out using mouth pipette technology. Visualization operations allow highly active single cells with complete morphologies to be accurately obtained. However, this process requires skilled operators and is subject to human interference, resulting in low throughput.

Microfluidic devices have been widely applied in single-cell isolation in the past few years due to their ability to isolate and manipulate single cells with microscale and integrated flow channels [21]. Microwell array chip-based methods [22,23] and droplet-based methods [24,25] are mainstream microfluidic-based single-cell isolation methods. Microfluidic-based methods have the advantages of high separation throughput and low cost, and they are easy to operate using commercial instruments. Based on these advantages, microfluidic-based technology is expected to become the best candidate tool for high-throughput single-cell isolation in the future. The characteristics of these single-cell isolation methods are shown in Table 1.

### 2.2. Single-Cell Lysis

After single-cell isolation, an appropriate single-cell lysis method is important for subsequent single-cell analyses. The aim of cell lysis is to obtain the single-cell DNA/RNA or protein, so adequate cell lysis is crucial for the accuracy of single-cell analysis. Major methods of single-cell lysis include chemical [26,27] and mechanical [28,29] methods. The former is relatively mild, while the latter is more intense, which is likely to cause DNA breakage in cells. Therefore, a suitable single-cell lysis method should be selected after considering several aspects, such as cell type, nucleic acid stability, and compatibility with downstream applications. Due to the difficulty of performing single-cell, and taking the downstream reaction into consideration, the chemical-lysis method is of top priority. After cell lysis, intra-cellular components of a single cell can be used for further analyses, such as proteomics, genomics, transcriptomics, and metabolomics.

## 3. Digital PCR

A digital polymerase chain reaction (dPCR) is an absolute quantitative analysis technology for nucleic acids, and it has high sensitivity and precision [30]. The reaction system is divided into a large number of independent micro reaction units for PCR amplification, with each unit containing 0 or 1 template. Then, the concentration of the initial sample can be calculated according to the positive fluorescence unit’s ratio and a Poisson statistical analysis. Compared to traditional PCR technology, digital PCR technology does not rely on the standard curve, has higher sensitivity and accuracy, and can realize the absolute quantitative analysis of a sample. Chamber-based digital PCR (cdPCR) [31,32,33] and droplet-based digital PCR (ddPCR) [34,35] are the two major methods of digital PCR. Among them, the method based on cdPCR is generally achieved through microfluidic chips with a large number of reaction chambers, and the method based on ddPCR utilizes droplets as the basic reaction unit.

**Figure 3:**
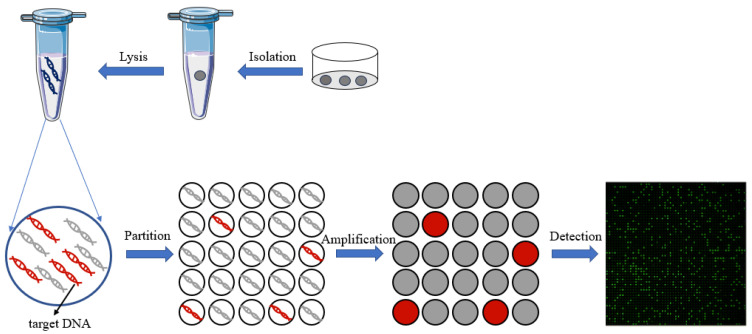
The principle of digital PCR for single-cell analysis.

In recent years, with the development of microfluidic technology, digital PCR has been widely used in gene mutation detection [36], copy number variation detection [37], virus microbial detection [38], genetically modified organisms’ detection [39,40], and other fields [41]. Compared to qPCR, digital PCR can achieve absolute quantification without standard curves, and it is an end-point-detection method which can increase detection sensitivity compared to qPCR reaction inhibition variations. On the other hand, the reaction units of dPCR are nanoliter chambers and droplets, which will help increase primer relative concentration to capture the low-abundance targets. Thus, dPCR technology can reduce the expression bias of different abundance targets in qPCR. All of these advantages make dPCR suitable for single-cell analysis. The principle of digital PCR for single-cell analysis is shown in Figure 3.

## 4. Digital PCR for Single-Cell Analysis

With the rapid development of single-cell sequencing technology, single-cell analysis has entered a new stage. However, to our knowledge, single-cell sequencing methods are very time-consuming and expensive for specific gene markers [42,43,44]. As mentioned above, with the development of microfluidic technology, digital PCR has been widely used in many fields. Nevertheless, in these research works, the digital PCR method is only used as a routine bulk diagnostic method and not for quantifying the single-cell gene expression. In this section, we summarize the recent advances in the application of digital PCR in single-cell analysis.

### 4.1. Chamber-Based Digital PCR (cdPCR) for Single-Cell Analysis

Chamber-based digital PCR (cdPCR) is one of the main methods commonly used for digital PCR [45]. Compared to droplet digital PCR (ddPCR), cdPCR has a more stable physical partition and is often simpler in operation. Therefore, it has promoted the application of digital PCR, and many cdPCR devices were developed for different purposes [46,47]. Next, we will introduce some studies based on cdPCR for single-cell analysis.

**Figure 4:**
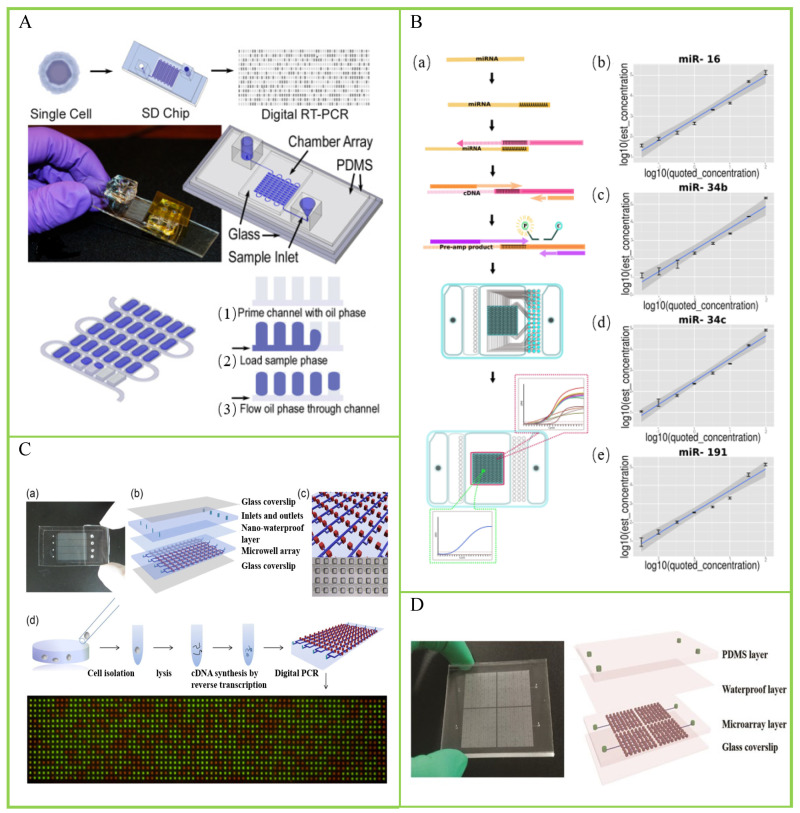
Chamber-based digital PCR (cdPCR) for single-cell analysis. (**A**) Components of the digital RT-PCR self-digitization chip. Reprinted from ref. [50]. (**B**) The microfluidics-based digital quantitative PCR (mdqPCR) workflow and proof-of-concept accuracy testing. (**a**) The mdqPCR workflow consists of three major steps: cDNA synthesis, template partition, and high-throughput qdPCR. (**b**–**e**) Standard curves showing correlations between quoted and test concentrations of four synthetic miRNAs including miR-16 (**b**), miR-34b (**c**), miR-34c (**d**), and miR-191 (**e**). Reprinted from ref. [51]. (**C**) Schematic drawing of the self-priming compartmentalization (SPC) digital PCR chip and procedure of single-cell digital PCR. (**a**) Photograph of the prototype of the SPC chip. (**b**) Schematic diagram of the layered device structure of the chip. (**c**) Diagram of the detail of the chip design. (**d**) The process of the single-cell gene expression digital PCR. Reprinted from ref. [53]. (**D**) Schematic drawings of the microfluidic chip for digital RT-PCR assays. Reprinted from ref. [55].

There are various types of RNA, from different biogenesis, and they possess distinct molecular characteristics. Traditional RNA analysis methods based on bulk cells cannot reflect the state of all cells or a group of cells in the sample. Studying RNA at the single-cell level can provide cell heterogeneity in tissues [48]. White et al. presented a high-throughput dPCR microfluidic device for the analysis of mRNA, micro-RNAs, and RNA editing events in single cells [49]. The cDNA from each single cell is distributed into a dedicated dPCR array consisting of 1020 independent 25 pL chambers, using surface-tension-based sample partitioning. They demonstrated its application in the absolute quantification of cDNA derived from mRNA and miRNA across over 1200 single cells. Then, they applied the chip-based single-cell dPCR to perform the measurements of single-nucleotide RNA editing of EEF2K in single K562 cells. Thompson et al. reported a self-digitization (SD) chip platform for the absolute quantification of single-cell mRNA [50], shown in Figure 4A. To validate the quantification ability of the SD-chip-based dPCR, the researchers used this method for absolute quantification of TFRC mRNA copies in a single cell and obtained similar results compared with single-molecule mRNA FISH assay. However, their method has one drawback. SD chips can only digitize 86.7% of the samples of single cells, resulting in sample loss. Yu et al. achieved a quantitative analysis of small RNAs at the single-cell level using digital quantitative PCR (dqPCR) technology based on the Fluidigm BioMark HD system [51]. The workflow is shown in Figure 4B. Moreover, they applied this method to miRNA analysis of single sperms and found that each sperm has a unique miRNA map, indicating that the small RNA carried by sperm may contribute to genetic and epigenetic diversity in offspring.

Cancer poses a serious threat to the health and safety of all humanity [52]; according to the latest estimated data from the International Agency for Research on Cancer (IARC) of the World Health Organization, there will be 19.29 million new cancer cases worldwide in 2021. Single-cell analysis has become a widely used tool in cancer research. It is used to characterize the cellular and molecular composition of tumors. Zhu et al. demonstrated a self-priming compartmentalization (SPC) chip for single-cell analysis with an 85% sample digitization [53]. The structure of the chip and the procedure of single-cell digital PCR are shown in Figure 4C. The researchers used the *β*-actin gene to assess the SPC chip’s ability, precision, and sensitivity for single-cell gene-expression analysis. Furthermore, they used an SPC digital chip to detect lung-cancer-related genes and PLAU gene expression of single A549 cells. The test results showed cellular heterogeneity among different A549 cells. To solve the sample-digitization-loss problem, they further developed a self-priming fractal branching microchannel net digital PCR chip with 100% sample digitization [54]. Based on this chip design, Xu et al. proposed a chip-based digital RT-PCR approach to investigate cancer-stem-cell-related marker genes at the single-cell level [55]. The structure of the chip is shown in Figure 4D. Cancer stem cells exist in cancer cells and are the culprits of cancer recurrence and metastasis. Due to its extremely rare presence, it is difficult to detect, and anticancer drugs also have difficulty in targeting them. Traditional research methods are mostly based on the strategy of bulk cells, and fail to recognize cellular heterogeneity. In this study, the researchers used single-cell digital RT-PCR to study four A549 cancer stem-cell-related marker genes simultaneously. The results showed that ALDH^+^ A549 cells in serum-free medium displayed a high expression of ALDH1A1 and ALCAM, which may help to discover new subpopulations and contribute to the classification and identification of CSCs. A major highlight of this work is that the samples of a single cell can achieve 100% digitization. Similarly, Chang et al. proposed a digital quantitative PCR (dqPCR) technology based on the PanelChip™ Analysis System to measure the epithelial–mesenchymal transition-gene expression levels in single A549 cells under TGFβ1 treatment [56]. The results showed that the expression levels of different marker genes showed different trends with the TGFβ1 treatment time.

Foodborne pathogens cause many diseases and pose serious economic and public health risks. As we know, even within a single strain, individual cells are also highly heterogeneous in gene expression. This is crucial for the survival of strains under specific conditions, such as bacterial persistence during antibiotic treatment [57]. Therefore, single-cell analysis of microorganisms is also very important. Liu et al. demonstrated a single-cell-based dPCR approach to the association of major Shiga toxin-producing *E. coli* (STEC) serogroups with major virulence genes [58]. This method can detect and confirm whether a given STEC serogroup carries a virulence gene in 1 day without cultural isolation or DNA extraction.

### 4.2. Droplet-Based Digital PCR (ddPCR) for Single-Cell Analysis

Droplet-based digital PCR (ddPCR) is another method commonly used for digital PCR, and ddPCR has been commercialized, such as Bio-Rad QX series and Naica crystal. Compared to cdPCR, it is simple to operate, has lower costs, and can achieve high-throughput detection [59]. In recent years, there has been some research on single-cell analysis based on ddPCR.

Mitochondria are the energy-producing organelle in cells and the main site for aerobic respiration. They are involved in various cellular functions, including energy conversion, tricarboxylic acid cycle, storage of calcium ions, regulation of membrane potential and control of programmed cell death, cell proliferation and metabolism, etc. Mitochondrial disease is a genetic disease that interferes with the production of energy in the body and is currently incurable. Single-cell mitochondrial analysis helps to understand heterogeneity dynamics and disease treatment. Maeda et al. developed a ddPCR method to determine mitochondrial heteroplasmy in a single cell [60]. Five strains of cells derived from patients diagnosed with mitochondrial disease were examined in this study to evaluate the heteroplasmy of mtDNA in a single cell. This method could reveal the existence of mtDNA variances at as low as 1% frequency in a single cell without cloning steps. O’Hara et al. presented a ddPCR method to measure mitochondrial DNA copy number in single-cells without requiring DNA purification or nuclear reference genes [61]. Changes in mitochondrial DNA copy numbers often accompanies diseases; thus, quantitation of mtDNA copy numbers in single cells is necessary to better understanding of the roles of mtDNA copy number changes in disease evolution. In this study, the researchers applied the ddPCR method to quantify mtDNA copy numbers in 73 single H1299 human lung cancer cells. The results showed significant variations in mtDNA copy number among different cells. Subsequently, they applied this method to study the changes in mtDNA levels in single T cells of different age groups after stimulation. The results showed that the mtDNA copy number peak was significantly higher in healthy centenarians than in frail ones. These data results suggest that mtDNA may be a potential biomarker of health status during aging. Similarly, Burr et al. presented a ddPCR-based protocol for measuring the mtDNA copy number in single cells from both human and mouse tissue [62]. In this work, the researchers proposed a standard protocol for single-cell analysis of mtDNA copy number, including primer and probe design, isolation of DNA from single cells, preparation of samples, generation of droplets, PCR, and droplet reading and analysis. Then, they applied this method to the serial measurement of the copy number in lysate from two individual mouse oocytes with 3.64% and 2.26% coefficients of variation (CV), respectively. Single-cell copy numbers of primary mouse primordial germ cells collected from four separate embryos at 13.5 days post-fertilization were measured to test for reproducibility across biological specimens.

**Figure 5:**
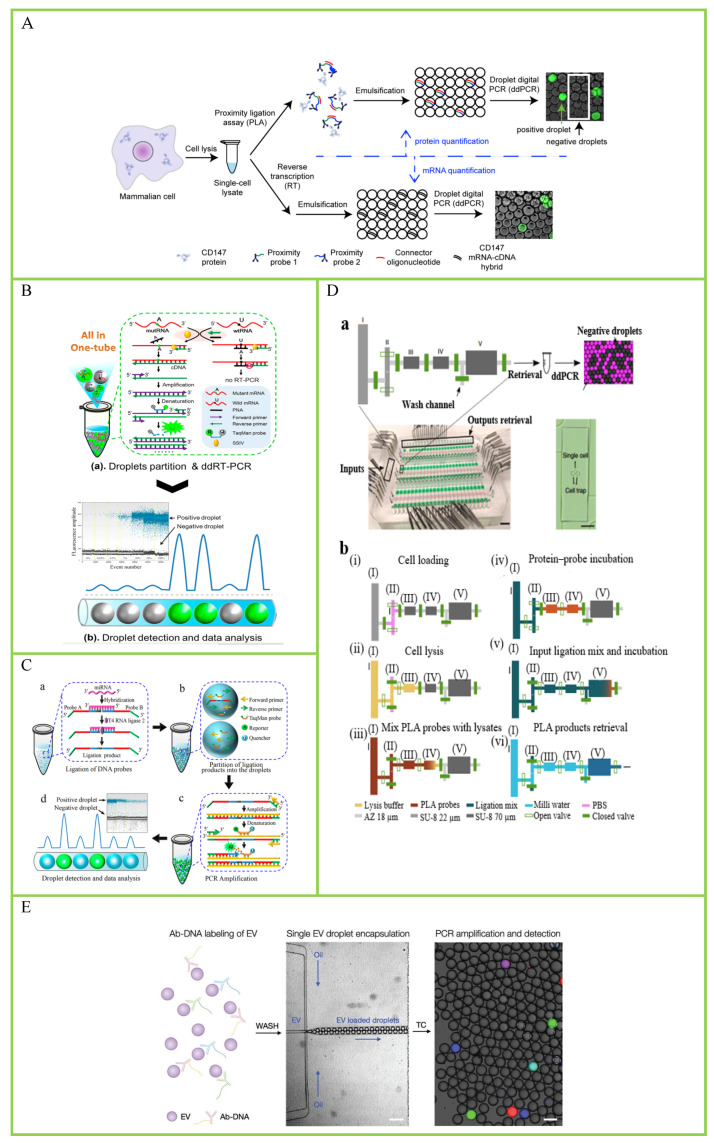
Droplet digital PCR (ddPCR) for single-cell analysis. (**A**) Digital PLA protocol for absolute protein and mRNA quantification from single cells. Reprinted from ref. [63]. (**B**) Illustration of the PNA clamp-based ddRT-PCR assay for the detection of mRNA mutation. Reprinted from ref. [65]. (**C**) Principle of the ddPCR-based miRNA assay. (**a**) Ligation of DNA probes. (**b**) Partition of the ligation products into the droplets. (**c**) PCR amplification. (**d**) Droplet detection and data analysis. Reprinted from ref. [66]. (**D**) Integrated microfluidic device for performing ultrasensitive single-cell protein/messenger RNA (mRNA) measurements. (**a**) Top: The schematic of one unit of assay chambers is shown; chamber sizes are not to scale. (I–V) represent different chambers. Bottom left: The chip image with food dye loaded in different channels, the scale bar is 2 cm. Bottom right: The microscope image of a single human embryonic kidney cell trapped in chamber II, the scale bar is 50 µm. (**b**) (i–vi) show the workflow of microfluidic-digital proximity ligation assay (µ-dPLA). Reprinted from ref. [64]. (**E**) Schematic of droplet-based single EV detection. Reprinted from ref. [69].

Messenger RNA (mRNA) are a large class of RNA molecules that transmits genetic information from DNA to the ribosome, where it serves as a template for protein synthesis. Absolute quantification of mRNA at the single-cell level helps to understand cellular heterogeneity and thus better understand the biological mechanism. Albayrak et al. presented a method by combining proximity ligation assay (PLA) and ddPCR to quantify proteins and mRNA simultaneously in single mammalian cells [63]. The dPCR protocol for absolute protein and mRNA quantification from single cells is shown in Figure 5A. In this work, the single-cell lysate is split into two: one is used to detect protein using digital PLA, and the other is used to detect mRNA using ddPCR. They applied the method to quantify endogenous CD147 proteins and mRNA copy numbers from the same individual human embryonic kidney (HEK293T) cells. Intriguingly, the results showed that the correlation between CD147 mRNA and protein is relatively low, while bulk-cells-based research found a significantly higher correlation. At last, a stochastic model of CD147 gene expression in single cells was constructed using this method. However, this method cannot quantify rare protein targets because of the large dilution factor introduced by sorting and lysing individual cells. Then, their research group improved the dPLA method and presented an automated microfluidic device for dPLA measurements (μ-dPLA) [64]. The integrated microfluidic device is shown in Figure 5D. They use this device to capture and sort individual cells into 7 nl chambers, dramatically reducing the dilution factor introduced using single-cell sorting and lysis steps. They applied this method to measure TNFR1 protein and mRNA from the same single cell (H1299) simultaneous with the duplex ddPCR reaction. The result shows increased single-cell mRNA–protein correlation.

Thereafter, Sun et al. proposed a robust one-step ddPCR-based method to quantify mRNA mutation in single cells [65]. The protocol of dPCR-based single-cell analysis is shown in Figure 5B. The researchers use a rationally designed peptide nucleic acid (PNA) to capture wild-type RNA without affecting the RT-PCR of mutant mRNA (mutRNA). Applying this strategy, they quantified mutRNA in three types of single cells including human melanoma cell line (SKMEL-28), cervical cancer cells (HeLa), and thyroid cancer cells (ARO). This method enables precise mRNA mutation detection in single cells with as low as 0.01% mutated mRNA in a high background of wild-type mRNA. MicroRNAs (miRNAs) are a class of endogenous small RNAs with a length of approximately 20–24 nucleotides, which play various important regulatory roles in cells. Therefore, the single-cell analysis of miRNAs at the single-cell level is crucial as it helps to better understand the relationship between miRNAs and cellular function. Tian et al. reported a ligation-based ddPCR method for miRNA detection in a single cell [66]. The principle of the ddPCR-based miRNA assay is shown in Figure 5C. In this work, two probes can be ligated with each other under the catalysis of T4 RNA ligase 2. Then, the researchers used this method to detect the let-7a miRNA in single human lung cancer cells (A549). The detection result showed that the copy numbers of let-7a in individual cells vary from 2293 to 4234, showing significant intercellular heterogeneity.

Telomerase is the basic nuclear protein reverse transcriptase, an enzyme responsible for telomere elongation in cells. Previous studies have shown that human telomerase reverse transcriptase is crucial for studying aging, stem cells, and cancer [67]. Due to the heterogeneity between cells, it is necessary to quantify telomerase activity in single cells. A. Ludlow et al. described a ddPCR-based method for quantitative telomerase enzyme activity determination in single cells [68]. The researchers used this method to detect telomerase activity in single HeLa cells, and the result showed that 57 of the 78 singles cells (73%) produced detectable signals above the background. This result indicates that telomerase activity exhibits cellular heterogeneity. Extracellular vehicles (EVs) are the collective term for vesicles released into extracellular space by cells. They play an important role in intercellular communication and participate in the regulation of a series of biological processes. Furthermore, they are ideal candidate biomarkers for diseases treatment. Ko et al. described an immuno-ddPCR-based method for single EV protein profiling [69]. A schematic of droplet-based single-EV detection is shown in Figure 5E. Then, they applied this method to profile PD-L1 expression of a single EV derived from different cancer cell lines. The result showed that Jurkat cells and their EV were strongly positive for CD4, and all three cell lines were negative for CD8 (control).

Ma et al. presented a novel microdroplet-based, one-step reverse-transcriptase polymerase chain reaction (RT-PCR) platform and demonstrated the detection of three targets simultaneously in a single experiment. And, they also demonstrated the detection of rare cell populations at a frequency of 0.1%. The multiplexity of this platform is a major highlight of this work [70].

## 5. Conclusions and Future Perspectives

Single-cell analysis helps to understand cellular heterogeneity, and single-cell data can provide a deeper understanding of biological processes. Many single-cell analysis methods have been developed in the past few years. This review summarized the main related work of digital PCR for single-cell analysis in the past decade. Chamber-based digital PCR and droplet-based digital PCR are the two main implementation methods with different advantages. Chamber-based digital PCR devices can achieve high-throughput single-cell analysis through a simple chip design. These chips have various structures with flexible operations and can achieve direct sample loading without external power sources, such as pumps. Physical partitioning in space is also a major advantage for multiple single-cell’s analyses. On the other hand, droplet-based digital PCR devices can generate a large number of droplets in a short period of time to achieve ultra-high-throughput single-cell analysis. Furthermore, these methods have relatively standardized processes due to the mature commercial digital PCR systems.

However, digital PCR for single-cell analysis also faces some urgent issues that need to be addressed. From the works summarized in this review, there is no standardized protocol of digital PCR for single-cell analysis. Many detection variations exist in terms of different methods, which will hinder the digital PCR to achieve large-scale applications for single-cell analysis. On the other hand, the whole process of single-cell analysis normally consists of a couple of steps, including single-cell isolation, single-cell lysis, and DNA/RNA amplification and analysis. So, the system complexity will significantly increase when more functional modules are integrated into a dPCR device for a higher level of automatic analysis. In the future research, a highly integrated, easy-to-operate, highly automated dPCR device with a standardized protocol is an important direction for single-cell analysis.

In this review, digital PCR for single-cell analysis have been applied to study cancer-related gene expression of single cells [53,55,56]. However, clinical research on cancer cells is still limited. In the future, clinical studies should be a key research direction of digital PCR for single-cell analysis. In contrast to the much greater multiplexity of protein detection on single cells using flow cytometry, the multiplexity of digital PCR for single-cell analysis is often restricted to specific applications. Ma et al. presented a microdroplet-based, multiplex RT-PCR platform and demonstrated the detection of three targets simultaneously [70]. In the future, the multiplexity of digital PCR for single-cell analysis should be given more attention by researchers. Due to the power of single-molecule-detection sensitivity of digital PCR, the single-cell digital PCR can be used to analysis many rare samples.

## Figures and Tables

**Table 1:** A summary of the main single-cell isolation methods mentioned in this review.

Method	Sample Type	Throughput	Cost	Isolation Efficiency	Automation	Reference
Limited serial dilution	Dissociated cell suspension	Low	Low	Low	No	[13]
FACS	Dissociated cell suspension	High	Very high	Low	Yes	[14]
Micromanipulation	Dissociated cell suspension	Low	Moderate	High	No	[15]
LCM	Tissue	Low	Moderate	High	No	[16]
Microfluidic	Dissociated cell suspension	Very high	High	Moderate	Yes	[17]

## Data Availability

Data are contained within the article.
